# An amino acid based system for CO_2_ capture and catalytic utilization to produce formates†

**DOI:** 10.1039/d1sc00467k

**Published:** 2021-03-02

**Authors:** Duo Wei, Henrik Junge, Matthias Beller

**Affiliations:** Leibniz-Institut für Katalyse e.V. Albert-Einstein-Str. 29a Rostock 18059 Germany henrik.junge@catalysis.de matthias.beller@catalysis.de

## Abstract

Herein, we report a novel amino acid based reaction system for CO_2_ capture and utilization (CCU) to produce formates in the presence of the naturally occurring amino acid l-lysine. Utilizing a specific ruthenium-based catalyst system, hydrogenation of absorbed carbon dioxide occurs with high activity and excellent productivity. Noteworthy, following the CCU concept, CO_2_ can be captured from ambient air in the form of carbamates and converted directly to formates in one-pot (TON > 50 000). This protocol opens new potential for transforming captured CO_2_ from ambient air to C1-related products.

## Abstract 2

A novel amino acid based reaction system for CO_2_ capture and utilization (CCU) to produce formates is presented applying a ruthenium-based catalyst. Noteworthy, CO_2_ can be captured from ambient air and converted to formates in one-pot (TON > 50 000).

## Graphical abstract

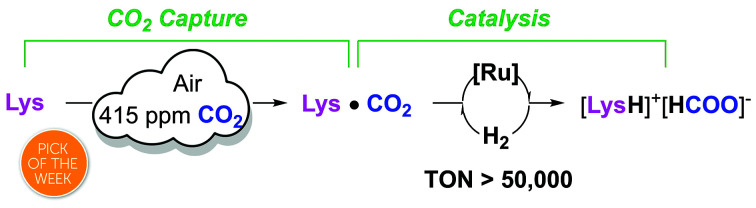

## Introduction

**Scheme 1:**
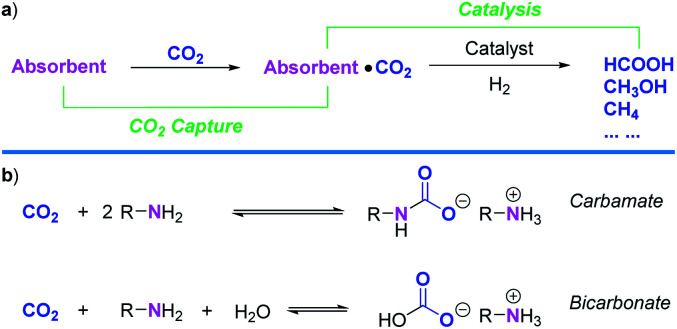
(a) Schematic CCU concept for CO_2_ hydrogenation to C1 products. (b) Reaction pathways for CO_2_ absorption with amines under aqueous conditions.

Carbon dioxide concentration in the atmosphere and global warming is ever-increasing with the enormous global energy demand supplied by consuming fossil fuels (mainly coal, oil, and natural gas).^1,2^ CO_2_ capture and storage (CCS) enable the use of fossil fuels with significantly lower CO_2_ emissions than usual.^3^ CCS is based on the separation of CO_2_ from energy conversion or other industrial processes, followed by compression, transport, and storage. However, CCS processes are meanwhile energy intensive as the electricity burden with amine scrubbing (113 kW h per mt CO_2_ removed) constitutes the minimum work to separate and compress CO_2_ (150 bar). Indeed, in two demonstration units, Boundary Dam and Thompsons, 210–220 kW h per mt were required for this purpose.^4^ Developing novel CO_2_ capture and utilization (CCU) methods for converting CO_2_ from air or flue gas not only saves energy from CCS (mainly CO_2_ desorption and compression steps) but also provides C1-related products (Scheme 1a).^5–12^ It's thus an important opportunity for developing a sustainable economy.^13–15^

In nature, inorganic carbon (particularly CO_2_) is converted to organic compounds by living organisms, which is known as carbon fixation, with photosynthesis as the most prominent example.^16^ It is estimated that approximately 258 billion tons of CO_2_ are converted into biomass by photosynthesis annually.^17^ As the most abundant protein on the Earth, ribulose 1,5-bisphosphate carboxylase/oxygenase (RuBisCO) is involved in the first major step of carbon fixation by plants and other photosynthetic organisms.^18,19^l-Lysine (Lys) is one of the six crucial amino acids (AAs) that are part of the active site of RuBisCO and it stabilizes CO_2_ in the form of carbamate for subsequent enzyme catalysis.^20^

By contrast, in industry *e.g.*, power plants, the most common process for capturing CO_2_ relies on the use of aqueous amine solutions (Scheme 1b).^3,21,22^ However, the maximum CO_2_ absorption capacity for an amine system varies based on which products are formed. When carbamates are the preferred products, this capacity is 50 mol% per amines at most. If bicarbonates are mainly formed, this capacity could reach up to 100 mol% per amines. Alkanolamines have been extensively investigated as chemical absorbents;^23^ however, their large-scale use also created some environmental concerns. Substituting such conventional amine absorbents with high boiling and innocuous natural AAs in combining CO_2_ capture and catalysis is therefore highly relevant. Noteworthy, CO_2_ capture with aqueous AAs,^24–27^ including Lys^25^ was already reported, but not its direct valorization. Based on the infusive phenomenon of carbon fixation by RuBisCO and our long-term interest in CO_2_ reduction, we report herein a CCU process which enables CO_2_ capture from ambient air and its conversion to formate in the presence of l-lysine. Moreover, to the best of our knowledge, there exists no example of catalytic hydrogenation of CO_2_ assisted by AAs.

**Fig. 1:**
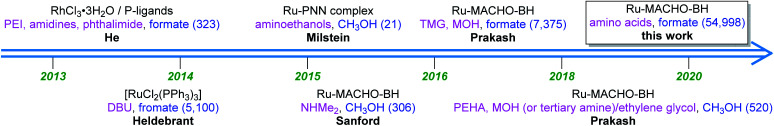
Representative catalysts and absorbents for CO_2_ hydrogenation to C1 products applied in CCU processes. The highest TON (turnover number) of formates or methanol are shown in parentheses, respectively.

Several Rh- and Ru-based homogeneous catalysts have been previously reported for CO_2_ capture and *in situ* hydrogenation to C1 products (Fig. 1).^8,28^ In 2013, pioneering work was performed by the group of He utilizing RhCl_3_·3H_2_O and phosphine ligands, for instance CyPPh_2_, DPEphos, and PPh_3_, as catalysts where gaseous CO_2_ was absorbed by polyethyleneimine (PEI),^29^ amidines,^30^ and potassium phthalimide^31^ as well as hydrogenated *in situ* to formates or formic acid.

In addition, ruthenium complexes have also been proven to be suitable catalysts for the hydrogenation of captured CO_2_ to formate or methanol. In 2014, Heldebrant and co-workers captured CO_2_ by DBU in methanol to form the methyl carbonate, which then was hydrogenated to formates catalyzed by [RuCl_2_(PPh_3_)_3_].^32^ One year later, Milstein and co-workers reported a CCU approach, where CO_2_ reacted with aminoethanols yielding oxazolidinones which were hydrogenated to CH_3_OH in 78–92% yield with a Ru-PNN pincer catalyst.^33^ In the same year, the Sanford group reported the CO_2_ capture with NHMe_2_ to form carbamate and subsequent hydrogenation to a mixture of DMF and CH_3_OH catalyzed by Ru-MACHO-BH complex.^34^ Employing the same catalyst and tetramethylguanidine (TMG),^35^ metal hydroxides,^36^ pentaethylenehexamine (PEHA),^37–39^ a mixture of metal hydroxides,^40^ or a tertiary amine^41^ with ethylene glycol as CO_2_ absorbent systems, Prakash and his colleagues combined CO_2_ capture from air with subsequent hydrogenation to produce formates or methanol. Recently, the group of Heldebrant reported a method where epoxides reacted with CO_2_ leading to cyclic carbonates. Then, *in situ* hydrogenation took place into methanol and glycol, with Ru-MACHO as catalyst.^42^

Compared to methanol, no hydrogen is lost in the form of water when formic acid or formate salts are produced by CO_2_ hydrogenation. Currently, formic acid is industrially produced by carbonylation of methanol to methyl formate and subsequent hydrolysis.^43^ It is mainly used as a preservative and antibacterial agent in livestock feed, *e.g.* silage and winter feed for cattle. In addition, formic acid is utilized in the production of leather and in dyeing and finishing textiles. More recently, it also gained interest as hydrogen storage medium as it contains 4.4 wt% of hydrogen with 53 g H_2_ per L of volumetric storage density.^7^

## Results and discussion

### CO_2_ capture with amino acids

For the development of a CCU concept to produce formic acid or formates, suitable CO_2_ absorbents must be used. Inspired by the carbon fixation pattern in nature, specifically RuBisCO, we considered applying AAs for this purpose.^24–27^ Thus, at the start of our investigations, we evaluated the ability of 12 different AAs, including the 6 ones involved in the active site of RuBisCo and some analogues to capture CO_2_. For this purpose, CO_2_ (2 bar) was charged into an aqueous solution of the respective AAs (5 M) and stirred at r.t. for 2–18 h.

As shown in Table S1,† most of the tested systems such as l-proline, l-glutamine, and l-histidine achieved only small to moderate amounts of CO_2_ capture, around 0.1 mol of CO_2_ per mol of AA (CO_2_/AA), (Table S1, entries 1–11†). Interestingly, in the presence of l-lysine (Lys), a significantly improved performance (3.63 mmol of captured CO_2_, corresponding to 0.73 CO_2_/Lys) was obtained in 18 h (Table S1, entry 12†). Such high CO_2_ capture efficiency could be attributed to the basic side chain of Lys, as its p*K*_a_ value is 10.7.

Thus, we investigated the effect of Lys for CO_2_ absorption under various conditions (Table 1 and Fig. S2 to S11†). As mentioned *vide supra*, carbon dioxide can be captured in form of Lys carbamates^26^ or Lys ammonium bicarbonate.^44^ Applying 20 bar of CO_2_, 0.83 CO_2_/Lys were obtained within only 0.5 h leading to carbamates and bicarbonate (ratio of 1 : 4, 98% total yield; Table 1, entry 1). A similar result was observed after 3 h (Table 1, entry 2). Also, at lower CO_2_ pressure (2 bar), significant absorption was achieved with 69–98% total yield of carbamates and bicarbonate within 0.5–18 h (Table 1, entries 3–5). Interestingly, in these cases (0.5 h and 3 h), mainly Lys carbamates were obtained. This shows that initially the corresponding carbamates are formed, which subsequently form bicarbonate.

**Table 1:** CO_2_ capture with Lys according to Scheme 1b under various conditions^a^

Entry	CO_2_ source	Time	Carbamates^b^ [mmol]	Bicarbonate^b^ [mmol]	Yield^c^ [%]	CO_2_/Lys^d^
1	CO_2_ (20 bar)	0.5 h	0.75	3.40	98%	0.83
2	CO_2_ (20 bar)	3 h	0.45	3.80	94%	0.85
3	CO_2_ (2 bar)	0.5 h	1.53	0.37	69%	0.38
4	CO_2_ (2 bar)	3 h	1.83	1.22	98%	0.61
5	CO_2_ (2 bar)	18 h	1.25	2.38	98%	0.73
6^e^	CO_2_ (2 bar)	3 h	1.18	n.d.	47%	0.24
7^f^	CO_2_ (2 bar)	3 h	0.30	n.d.	12%	0.06
8	Air	1 d	1.40	n.d.	56%	0.28
9	Air	2 d	1.95	n.d.	78%	0.39
10	Air	4 d	2.42	n.d.	97%	0.48
11	Air	8 d	2.45	n.d.	98%	0.49
12^g^	Air	4 d	8.20	n.d.	82%	0.41

a Conditions: Lys (5.0 mmol), H_2_O (1.0 mL), stirred at r.t. Air bubbling: 1 L min^−1^.

b Determined by ^13^C NMR-quant with THF (406.2 μL, 5.0 mmol) as internal standard.

c Total yield of carbamates and bicarbonate based on Lys.

d Mols of CO_2_ captured per mol of Lys.

e THF (1 mL) as solvent.

f Neat condition (without solvent).

g Lys 20.0 mmol. n.d. = not detectable. Experiments were performed at least twice; average values are used (st. dev. < 10%).

Besides water, the aprotic solvent THF was applied. After 3 h exclusively the carbamate was formed (1.18 mmol corresponding to 0.24 CO_2_/Lys, Table 1, entry 6). A much lower CO_2_/Lys ratio (0.06) was observed under neat conditions (without solvent, Table 1, entry 7). Next, to demonstrate the viability of our general CCU methodology, ambient air, containing *ca.* 415 ppm (parts per million) CO_2_, was bubbled through Lys solution for 1–8 days (Fig. S1†). Indeed, up to 0.49 mol CO_2_ per mol Lys were absorbed representing a yield of 98% with carbamates as sole products. Performing the reaction on multi-g scale (20 mmol Lys), 8.20 mmol CO_2_ were captured corresponding to 0.41 CO_2_/Lys and 82% carbamate yield (Table 1, entry 12).

### Catalytic hydrogenation of CO_2_ to formate

Next, to identify a suitable reduction system, various metal pincer complexes were tested for the hydrogenation of gaseous CO_2_ in the presence of different amino acids (Tables 2 and S2, Fig. S12 and S13†). To our delight, testing the Ru-MACHO-BH complex (**Ru-1**, 0.2 mol%) in H_2_O/THF (1 : 1 mixture) revealed significant activity in the presence of Lys for the hydrogenation of gaseous CO_2_ to formate (71% yield based on Lys) at 145 °C (Table S2, entry 1†).

**Table 2:** Ru-catalyzed hydrogenation of gaseous CO_2_ in the presence of Lys^a^

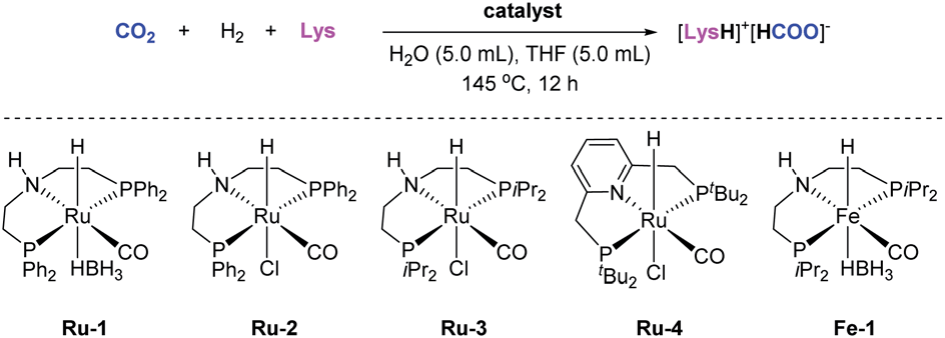			
Entry	Cat. [μmol, ppm]	Formate^b^ [mmol]	% Yield^c^ (TON)^d^
1	**Ru-1** [2.0, 400]	4.37	87 (2187)
2	**Ru-1** [0.2, 40]	3.89	78 (19 440)
3	**Ru-1** [0.02, 4]	3.95	79 (197 559)
4	**Ru-2** [0.02, 4]	4.24	85 (212 139)
5	**Ru-2** [0.01, 2]	1.48	30 (147 906)
6^e^	**Ru-1** [0.02, 4]	2.77	55 (138 510)
7^e^	**Ru-2** [0.02, 4]	2.90	58 (144 990)
8^e^	**Ru-3** [0.02, 4]	0.29	6 (14 580)
9^e^	**Ru-4** [0.02, 4]	2.35	47 (117 450)
10^e^	**Fe-1** [0.02, 4]	n.d.	—

a Conditions: catalyst, Lys (5.0 mmol), H_2_O (5.0 mL), THF (5.0 mL), CO_2_ (20 bar), H_2_ (60 bar), 145 °C, 12 h.

b Determined by ^1^H NMR with DMF (250 μL, 3.24 mmol) as internal standard.

c Calculated by formate [mmol]/Lys [mmol].

d Calculated by formate [mmol]/catalyst [mmol].

e 3 h. n.d. = not detectable. Experiments were performed at least twice; average values are used (st. dev. < 10%).

On the other hand, l-cysteine, l-histidine, l-serine, and l-threonine led to formates in much lower yields (up to 13%), while other AAs, such as glycine, l-proline, and l-glutamine showed no activity at all in the presence of catalyst **Ru-1**, (Table S2, entries 2–12†). Taking Lys as a benchmark CO_2_ absorbent, the TON of formate can be considerably increased from 2187 to 197 559 when decreasing the loading of **Ru-1** from 400 ppm (based on Lys) to 4 ppm (Table 2, entries 1–3). With 4 ppm of Ru-MACHO (**Ru-2**) as catalyst, the highest TON 212 139 was achieved (Table 2, entry 4). Interestingly, in these reactions, CO_2_ was selectively converted to formate in up to 87% yield with less than 1% of formamide. Next, several ruthenium pincer complexes were tested at 4 ppm loading for the hydrogenation of gaseous CO_2_ in the presence of Lys within 3 h (Table 2, entries 6–10). **Ru-1** and **Ru-2** gave formate in 55% and 58% yields, respectively, whereas Ru-MACHO^iPr^ (**Ru-3**) was less active leading to formate in only 6% yield. With Milstein's Ru-PNP complex (**Ru-4**) as catalyst, formate was obtained in 47% yield. However, no formate can be detected in the reaction catalyzed by Fe-MACHO^iPr^-BH complex (**Fe-1**).

Several blank reactions were also carried out (Table S3†): in the absence of either Lys, **Ru-1**, or CO_2_, no formate was detectable. These results clearly demonstrate that Lys and **Ru-1** are both crucial to promote the hydrogenation of CO_2_ from air to formate. Reactions with other solvents, for example, triglyme, methanol, ethylene glycol or their 1 : 1 mixture with water could not improve the reaction efficiency (Table S4†). When replacing THF with the more eco-friendly green solvent 2-methyltetrahydrofuran (2-MTHF),^45^ a comparable yield of formate (86%) was observed. Lowering the temperature from 145 to 105 °C, the yield of formate decreased only slightly from 79% to 64% (Table S5†).

### Development of a general CCU concept

After having studied the individual processes of (a) CO_2_ absorption and (b) CO_2_ reduction in the presence of Lys, the overall CCU concept was demonstrated by combining CO_2_ capture and *in situ* hydrogenation to formate (Table 3 and Fig. S14–S16†).

**Table 3:** Combining CO_2_ capture from ambient air and *in situ* conversion to formate^a^

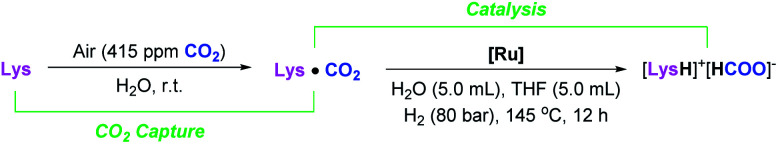				
Entry	Captured CO_2_ [mmol]	Cat. [μmol]	Formate^b^ [mmol]	% Yield^c^ (TON)^d^
1	2.42	**Ru-1** [2.0]	1.10	46 (551)
2	2.42	**Ru-1** [0.85]	1.15	48 (1353)
3	2.42	**Ru-1** [0.17]	1.02	42 (6004)
4	2.42	**Ru-1** [0.02]	1.10	45 (54 998)
5	2.42	**Ru-2** [0.02]	1.04	43 (52 245)
6^e^	8.20	**Ru-1** [0.08]	2.40	29 (29 993)
7^e^	8.20	**Ru-2** [0.08]	3.31	40 (41 330)
8^e^	8.20	**Ru-2** [0.04]	1.00	12 (25 110)

a Conditions: CO_2_ captured from air within 4 d applying 5 mmol Lys, given amount of catalyst dosed from stock solution, H_2_O (5.0 mL), THF (5.0 mL), H_2_ (80 bar), 145 °C, 12 h.

b Determined by ^1^H NMR with DMF (250 μL, 3.24 mmol) as internal standard.

c Calculated by formate [mmol]/captured CO_2_ [mmol].

d Calculated by formate [mmol]/catalyst [mmol].

e CO_2_ captured with 20 mmol Lys. Experiments were performed at least twice; average values are used (st. dev. < 10%).

Using captured CO_2_ (2.42 mmol) as substrate in the presence of **Ru-1** (2.0 μmol) as catalyst, 46% formate yield (based on captured CO_2_) was obtained (TON 551; Table 3, entry 1). The highest TON reached 54 998 with 0.02 μmol **Ru-1**, while the yield was maintained at 45% (Table 3, entries 2–4). **Ru-2** showed comparable activity for the hydrogenation of captured CO_2_ yielding 43% of formate (Table 3, entry 5). With 8.20 mmol captured CO_2_, 29% of formate were obtained with **Ru-1** at 0.08 μmol loading (Table 3, entry 6). 3.31 mmol formate (40% yield) were obtained with the same amount of **Ru-2** (Table 3, entry 7).

**Fig. 2:**
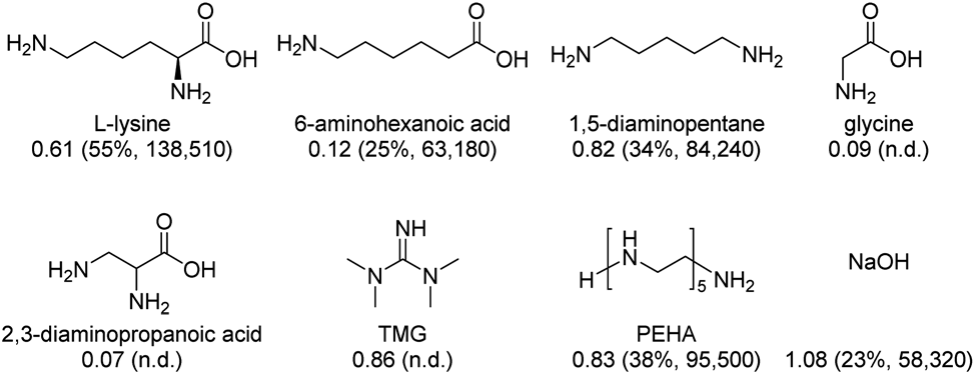
Various Lys analogues and benchmark amines applied in the CO_2_ absorption and hydrogenation processes performed under conditions in Table 1, entry 4 and Table 2 entry 6, respectively. CO_2_/amine (mols of CO_2_ captured per mol of amine) are shown with yield and TON of formates in parentheses; n.d. = not detectable.

Finally, some Lys analogues and derivatives as well as selected benchmark amines^35,37,39^ were applied according to our overall protocol (Fig. 2). In the presence of 6-aminohexanoic acid and 1,5-diaminopentane, 0.12 and 0.82 CO_2_/amine were achieved and formates were obtained in yields of 25% and 34%, respectively. Noticeably, 2,3-diaminopropanoic acid and the simplest amino acid glycine did not show any activity in both CO_2_ absorption and hydrogenation processes. In the case of TMG and PEHA, CO_2_ was captured with 0.86 and 0.83 CO_2_/TMG or PEHA, respectively. However, the presence of TMG inhibited the hydrogenation of CO_2_, whereas PEHA led to formate and formamides in 38% and 8% yield, respectively. Applying the inorganic base NaOH^36^ resulted in a CO_2_/base ratio of 1.08 and 23% formate yield. All these experiments demonstrate the superiority of using Lys for carbon dioxide capture and direct valorizations. It also indicates the crucial presence of an α-amino acid moiety and an additional amine function in the side chain of AA.

To rationalize the perfect selectivity towards formates in the current study, we conducted further experiments by heating up the mixture of formic acid and Lys or PEHA in H_2_O at 145 °C (Table S6†). Indeed, Lys led to formate in quantitative yield without any formamide detectable after 12 h, whereas PEHA gave 28% yield of formamide along with 71% formate. Obviously, the less basic conditions applying Lys (pH 10.2 for a 5 M aqueous solution) prevented the formation of formamides taking place in the presence of PEHA (pH 13.4).

## Conclusions

In conclusion, we described an amino acid based catalyst system for the highly relevant CO_2_ capture and utilization (CCU) process to produce formates in one-pot. The naturally occurring amino acid l-lysine affords formate generation with a high efficiency. Among the investigated catalysts, the most active ones are identified with Ru-MACHO complexes (**Ru-1** and **Ru-2**) for the hydrogenation of gaseous CO_2_ (TON > 210 000) and the *in situ* hydrogenation of captured CO_2_ (TON > 50 000). Noteworthy, in the present CCU concept, CO_2_ can be captured from ambient air in the form of carbamates and hydrogenated to formate directly.

## Author contributions

D. W. conducted all the experimental work, collected and analyzed the data. D. W., H. J. and M. B. wrote the paper. H. J. and M. B. proposed and supervised the project. All the authors discussed the results and commented on the manuscript. All authors have given approval to the final version of the manuscript.

## Conflicts of interest

There are no conflicts to declare.

## Supplementary Material

SC-012-D1SC00467K-s001
