# Supplementary material for: An amino acid based system for CO2 capture and catalytic utilization to produce formates
Source: Chem Sci. 2021 Mar 2;12(17):6020–4. doi: 10.1039/d1sc00467k (PMC8098692; doi:10.1039/d1sc00467k)
Supplement: SC-012-D1SC00467K-s001 [file SC-012-D1SC00467K-s001.pdf]

Electronic Supplementary Material (ESI) for Chemical Science.
This journal is © The Royal Society of Chemistry 2021

Supporting Information

An amino acid based system for CO_2 capture and catalytic utilization to produce formates

Duo Wei, Henrik Junge,* and Matthias Beller*

Leibniz-Institut für Katalyse
Albert-Einstein-Straße 29a, 18059 Rostock (Germany)
E-Mail: henrik.junge@catalysis.de; matthias.beller@catalysis.de

Contents

Materials and methods 1

CO_2 capture with amino acids 1

Standard procedure for the hydrogenation of gaseous CO_2 9

Standard procedure for CO_2 capture from ambient air and *in situ* conversion to formate. 14

Reference 16

Materials and methods

Unless otherwise stated, all reactions were conducted under an argon atmosphere. Ru-MACHO-BH (**Ru-1**, Strem, 98%), Ru-MACHO (**Ru-2**, Strem, 98%), Ru-MACHO^{iPr} (**Ru-3**, Strem, 97%), L-lysine (TCI, 98%), L-tyrosine (TCI, >98.5%), L-threonine (TCI, >99%), L-methionine (Alfa Aesar, >98%), L-glutamic acid (TCI, >99%), L-serine (TCI, >99%), L-proline (Acros Organics, >99%), L-cysteine (TCI, >98%), L-histidine (Sigma-Aldrich, >99%), L-tryptophan (TCI, >98.5%), glycine (Merck, >99.7%), L-glutamine (TCI, >99%), 1,5-diaminopentane (TCI, >98%), 6-aminohexanoic acid (Alfa Aesar, 99%), 2,3-diaminopropanoic acid (fluorochem, 95%), tetramethylguanidine (Alfa Aesar, 99%), pentaethylenehexamine (Sigma-Aldrich, >98%), deuterium oxide (Deutero, 99.9%) were purchased from commercial suppliers and used without further purification. Milstein's Ru-PNP complex (**Ru-4**)[1] and Fe-MACHO^{iPr}-BH (**Fe-1**)[2] were synthesized according to literature. ^{1}H and ^{13}C were recorded using Bruker AV 300 MHz and Bruker AV 400 MHz spectrometers. ^{1}H and ^{13}C NMR chemical shifts were determined relative to the internal standard THF (3.74 ppm and 68.68 ppm respectively) or DMF (7.92 ppm and 165.53 ppm respectively) in D_2O. ^{13}C NMR-quant were performed with relaxation delay = 20s (rd>20s did not change the integration), number of scans = 512, acquisition time = 1.1141s.[3] Deionized (DI) water was used for CO_2 capture and hydrogenation reactions.

CO_2 capture with amino acids

Capture with CO_2 (2 bar): Amino acid (5.0 mmol) was added in a 25 mL Schlenk tube, followed with 1.0 mL of DI water, then 2 bar of CO_2 was charged into the Schlenk. Afterwards, the Schlenk tube was closed and stirred at r.t. for 2-18 h. The captured CO_2 amounts were calculated by gravimetric analysis.

Table S1. CO_2 capture with amino acids under 2 bar of CO_2.

Entry	AA [5 M]		Time [h]	Captured CO_2 [mmol][a]	CO_2/AA[b]
1	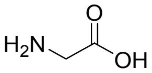	glycine	2<br>18	0.47<br>0.47	0.09<br>0.09
2	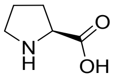	L-proline	2<br>18	0.39<br>0.40	0.08<br>0.08
3	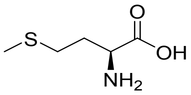	L-methionine	2<br>18	0.27<br>0.28	0.05<br>0.06
4	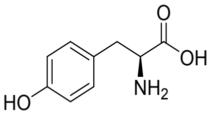	L-tyrosine	3<br>18	0.45<br>0.47	0.09<br>0.09
5	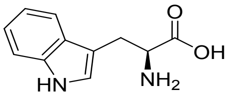	L-tryptophan	3<br>18	0.26<br>0.30	0.05<br>0.06
6	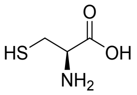	L-cysteine	3<br>18	0.54<br>0.61	0.11<br>0.12
7	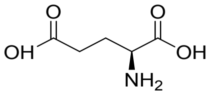	L-glutamic acid[c]	2<br>18	0.44<br>0.45	0.09<br>0.09
8	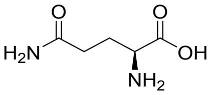	L-glutamine[c]	2<br>18	0.24<br>0.26	0.05<br>0.05
9	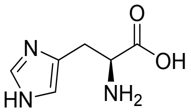	L-histidine[c]	3<br>18	0.41<br>0.72	0.08<br>0.14
10	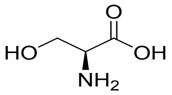	L-serine[c]	3<br>18	0.38<br>0.54	0.08<br>0.11
11	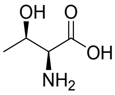	L-threonine[c]	2<br>18	0.46<br>0.49	0.09<br>0.10
12	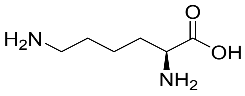	L-lysine[c]	3<br>18	3.05<br>3.63	0.61<br>0.73
13	None		2<br>18	n.d.<br>n.d.	-<br>-

Conditions: AA (5.0 mmol), H_2O (1.0 mL), CO_2 (2 bar), stirred at r.t. [a] Calculated by gravimetric analysis [b] mols of CO_2 captured per mol of AA. [c] AAs involved as RuBisCO active site. n.d.= not detectable. Experiments were performed at least twice; average values are used (St. Dev.<10%).

Capture with CO_2 (20 bar): L-lysine (5.0 mmol) was added in a 50 mL autoclave equipped with a magnetic stir bar, followed with 1.0 mL of DI water, then 20 bar of CO_2 was charged into the 50 mL autoclave. Afterwards, the autoclave was closed and stirred at r.t. for 0.5-3 h. The captured CO_2 amounts were calculated by ^{13}C NMR-quant with THF (406.2 μL, 5.0 mmol) as internal standard.[3]

Capture from ambient air: L-lysine (5.0 mmol) was added in a 25 mL vial followed with 15.0 mL of DI water, then the indoor air (containing ca. 400 ppm CO_2) was bubbled through the vial using a long needle (1 L/min.). After 4 days, the amount of the solvent reduced to ca. 1 mL due to the water evaporation. THF (406.2 μL, 5.0 mmol) was added as an internal standard to the solution, and the mixture was analyzed by ^{13}C NMR-quant.[3]

**L-lysine (5.0 mmol scale)**		
Before air bubbling (1 mL solution)	Before air bubbling (15 mL solution)	After air bubbling 4 days (1 mL solution)
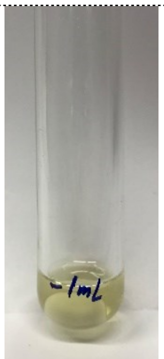	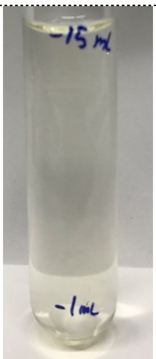	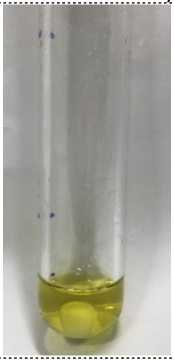
**L-lysine (20.0 mmol scale)**		
Before air bubbling (5 mL solution)	Before air bubbling (15 mL solution)	After air bubbling 4 days (5 mL solution)
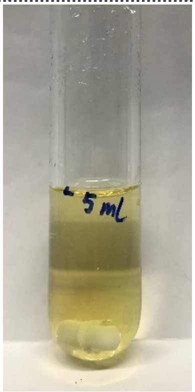	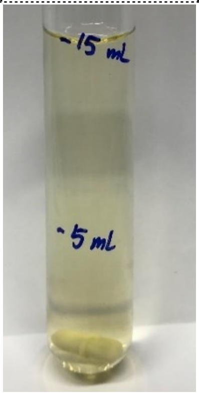	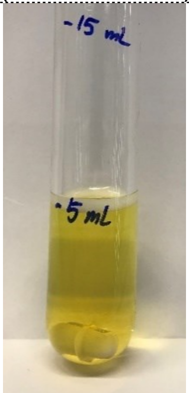

Figure S1. Typical reaction mixture of CO_2 capture from ambient air with L-lysine.

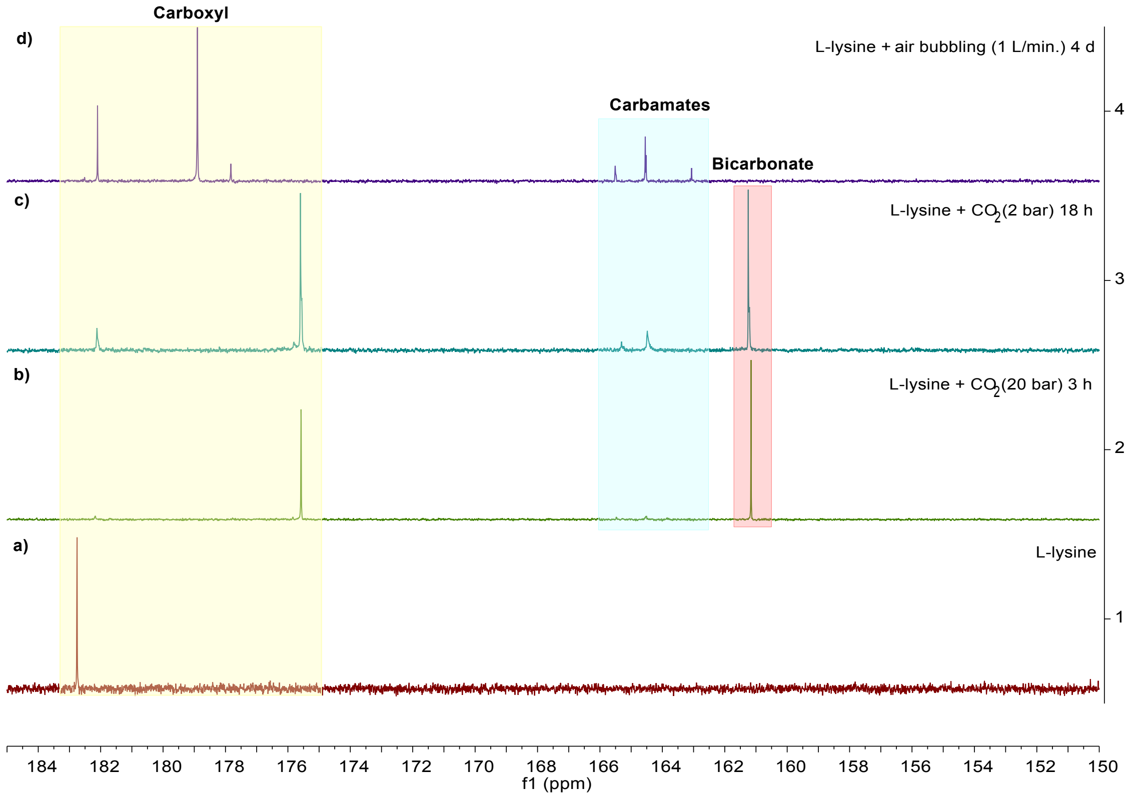

Figure S2. ^{13}C NMR-quant (185 - 150 ppm) in D_2O of **a)** L-lysine and corresponding solution after CO_2 capture with **b)** 20 bars of CO_2 (3 h), **c)** 2 bars of CO_2 (18 h) and **d)** air bubbling (1 L/min.) 4 d.

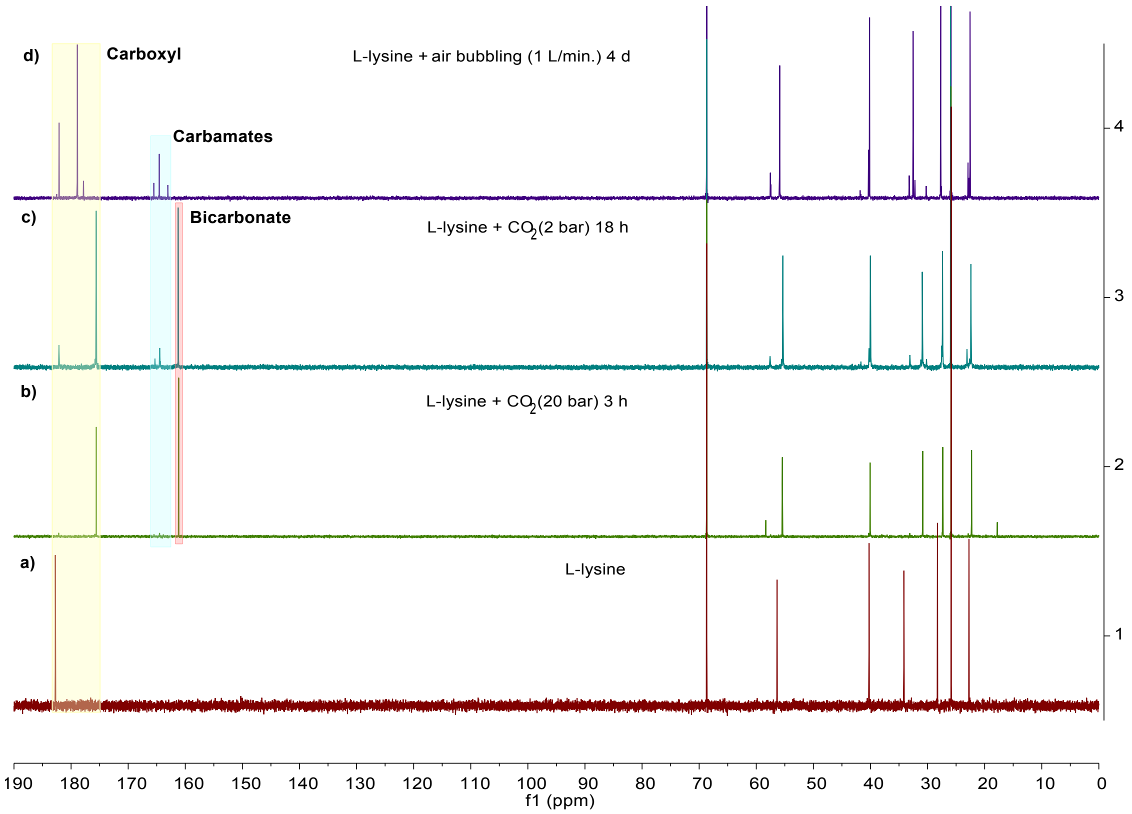

Figure S3. ^{13}C NMR-quant (190 - 0 ppm) in D_2O of **a)** L-lysine and corresponding solution after CO_2 capture with **b)** 20 bars of CO_2 (3 h), **c)** 2 bars of CO_2 (18 h) and **d)** air bubbling (1 L/min.) 4 d.

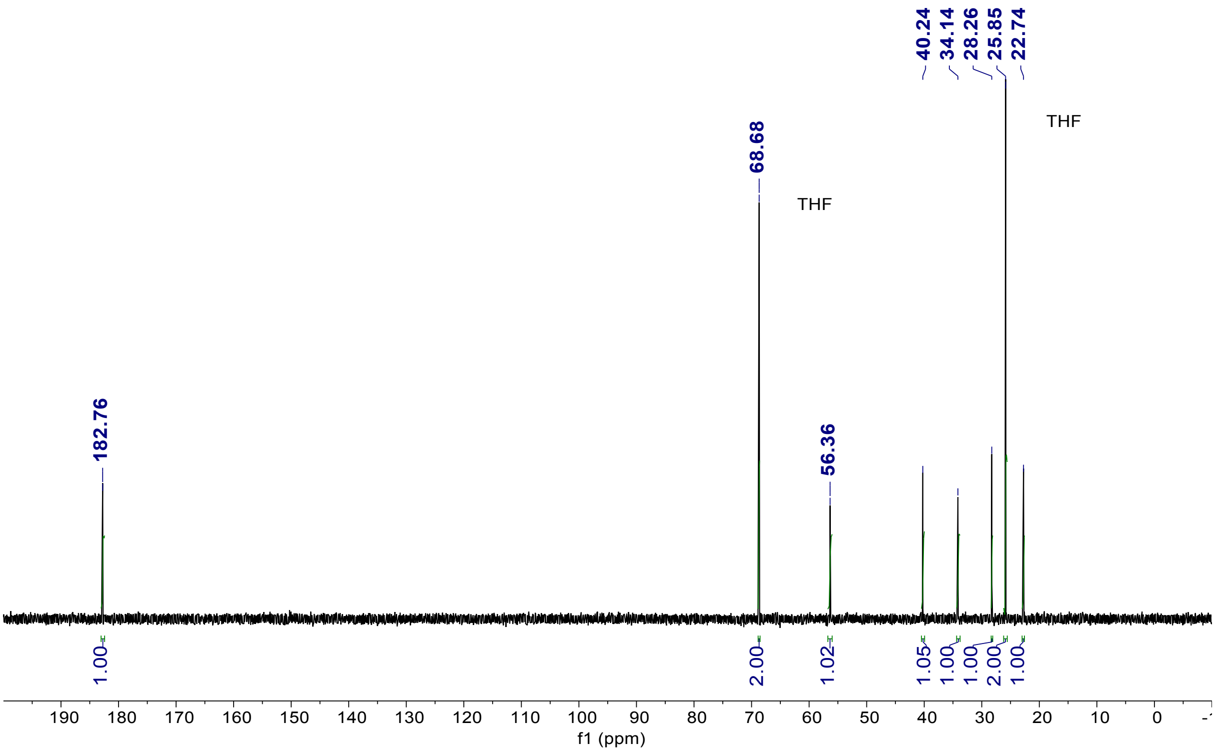

Figure S4. ^{13}C NMR-quant of L-lysine in D_2O.

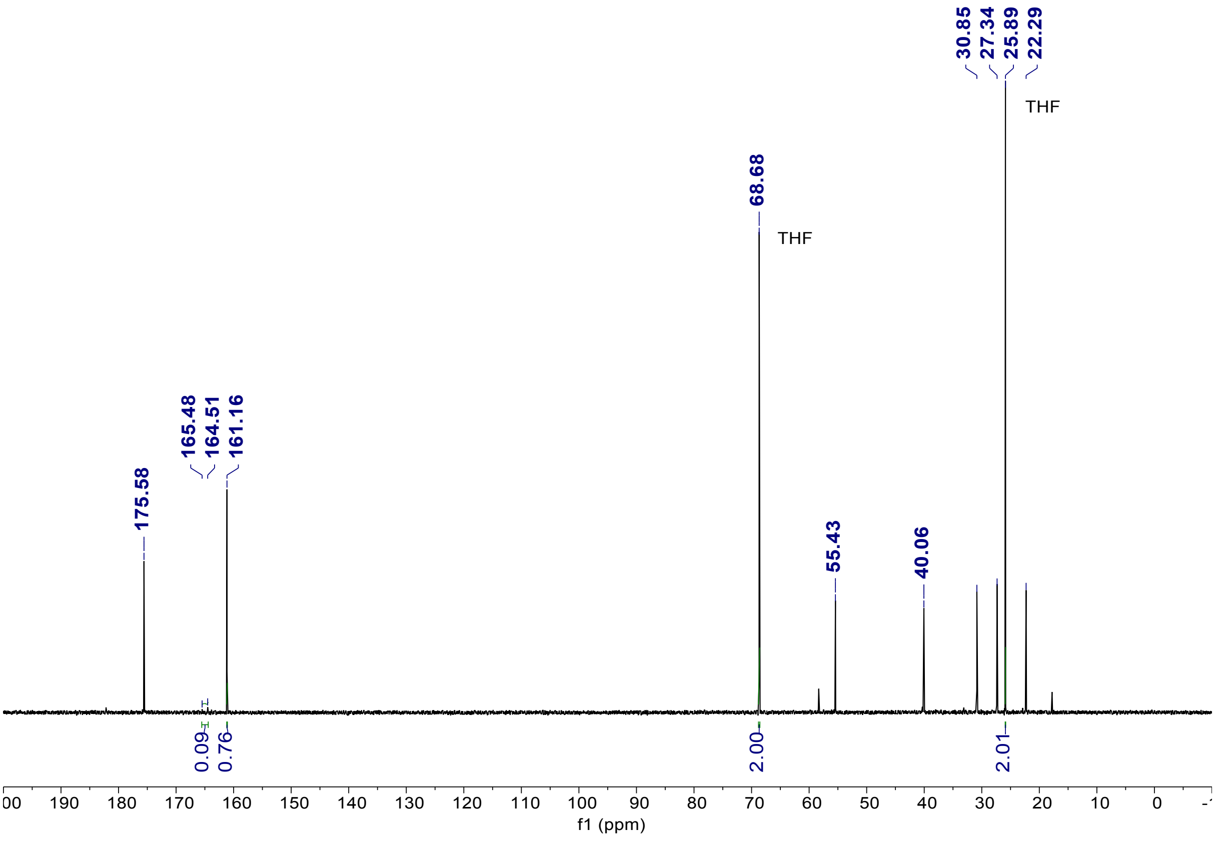

Figure S5. ^{13}C NMR-quant in D_2O of CO_2 capture under 20 bars of CO_2 (3 h) with 5.0 mmol L-lysine.

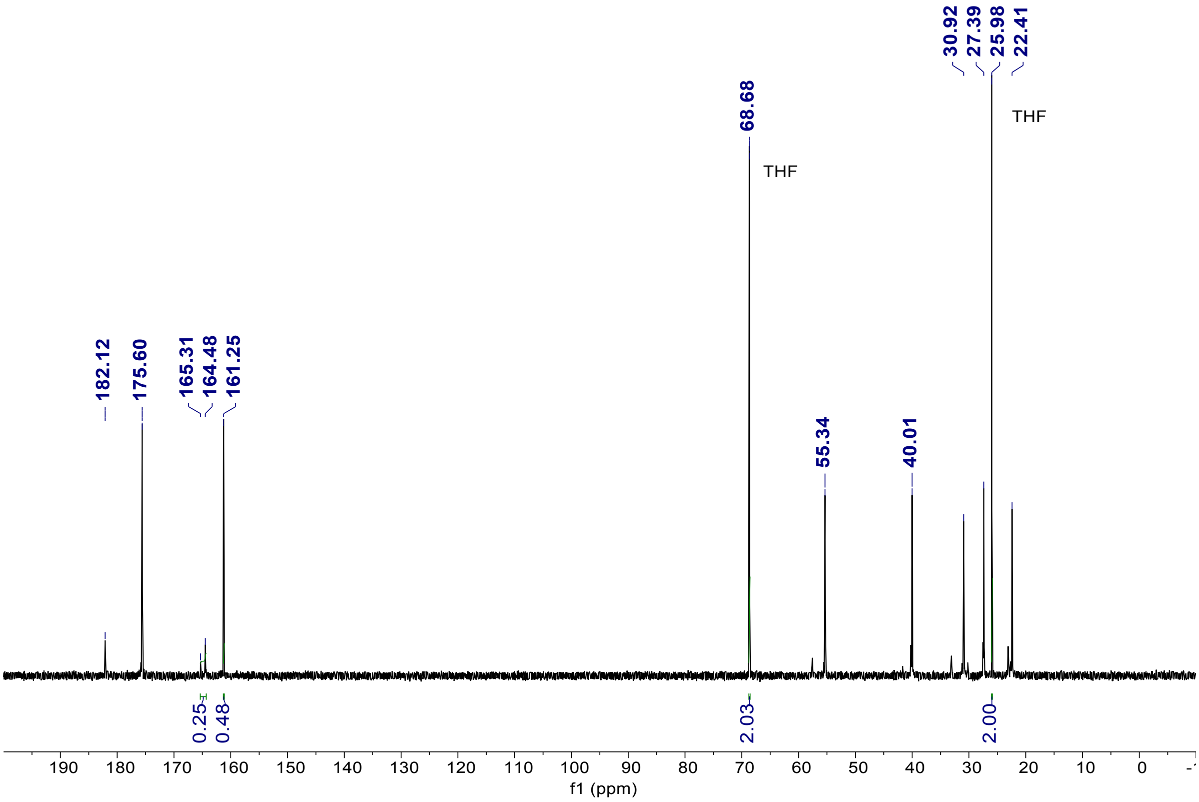

Figure S6. ^{13}C NMR-quant in D_2O of CO_2 capture under 2 bars of CO_2 (18 h) with 5.0 mmol L-lysine.

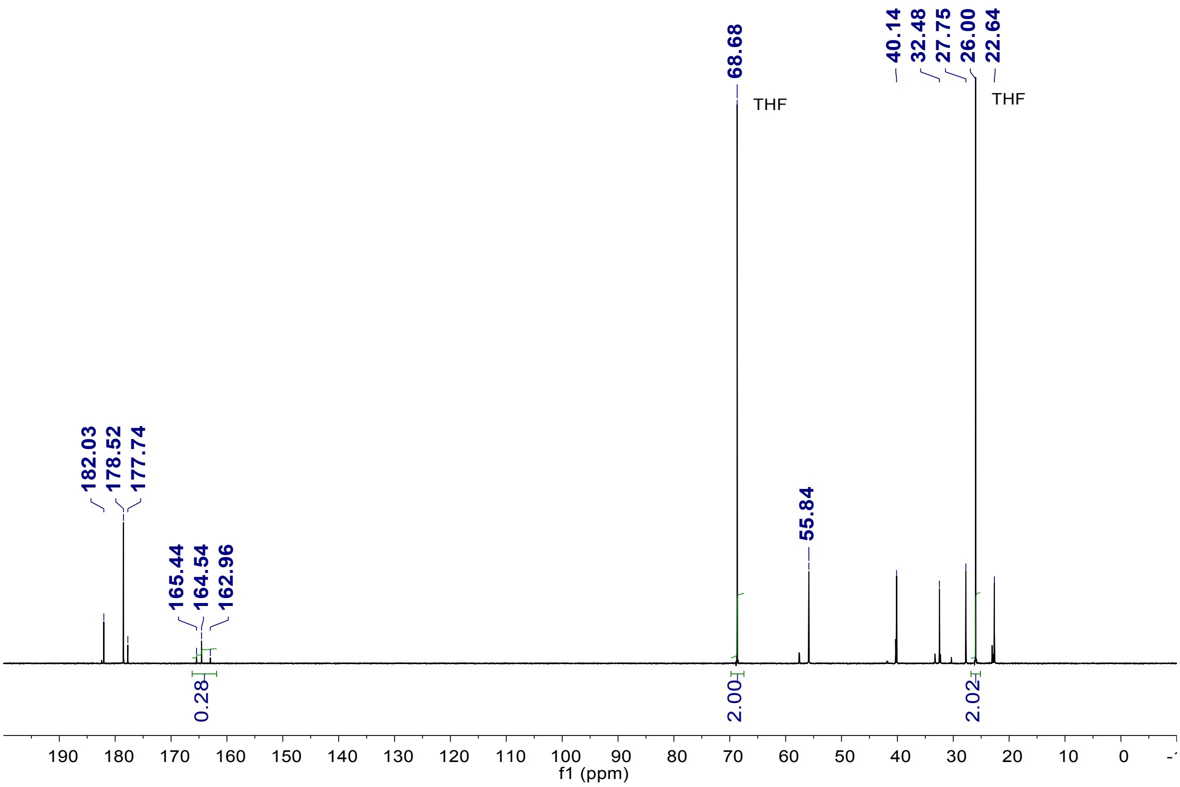

Figure S7. ^{13}C NMR-quant in D_2O of CO_2 capture with air bubbling (1 L/min. 1 day) with 5.0 mmol L-lysine.

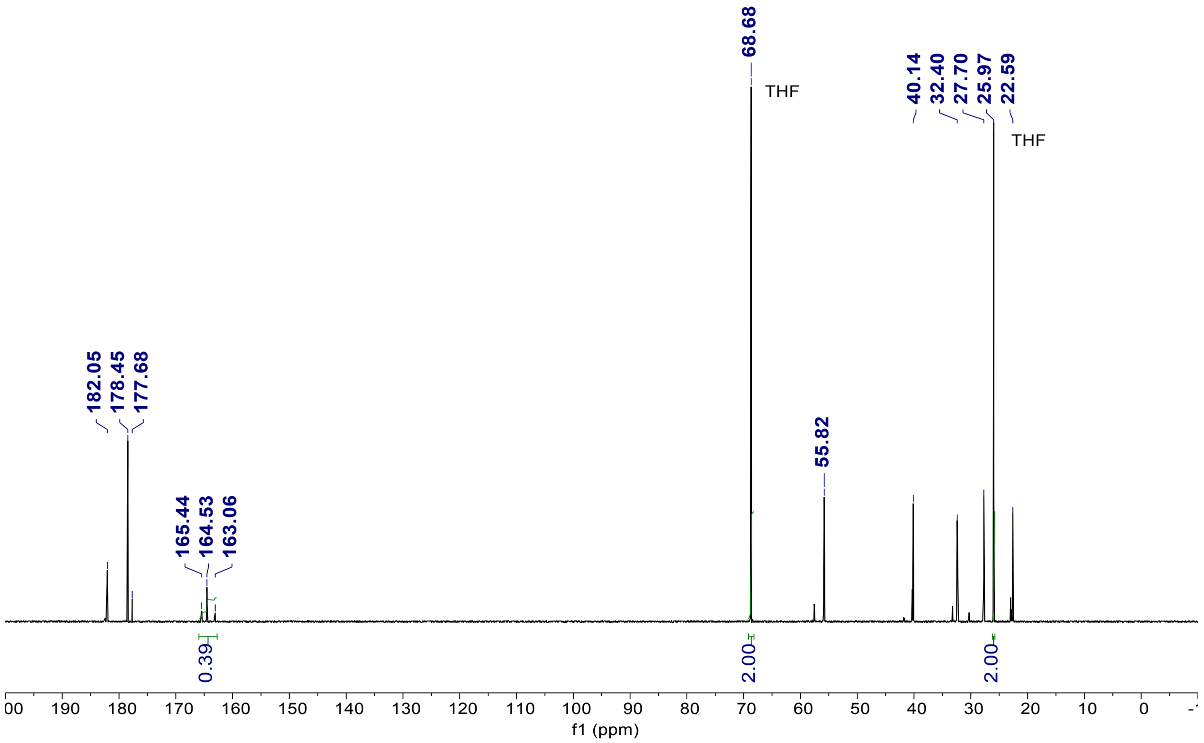

Figure S8. ^{13}C NMR-quant in D_2O of CO_2 capture with air bubbling (1 L/min. 2 days) with 5.0 mmol L-lysine.

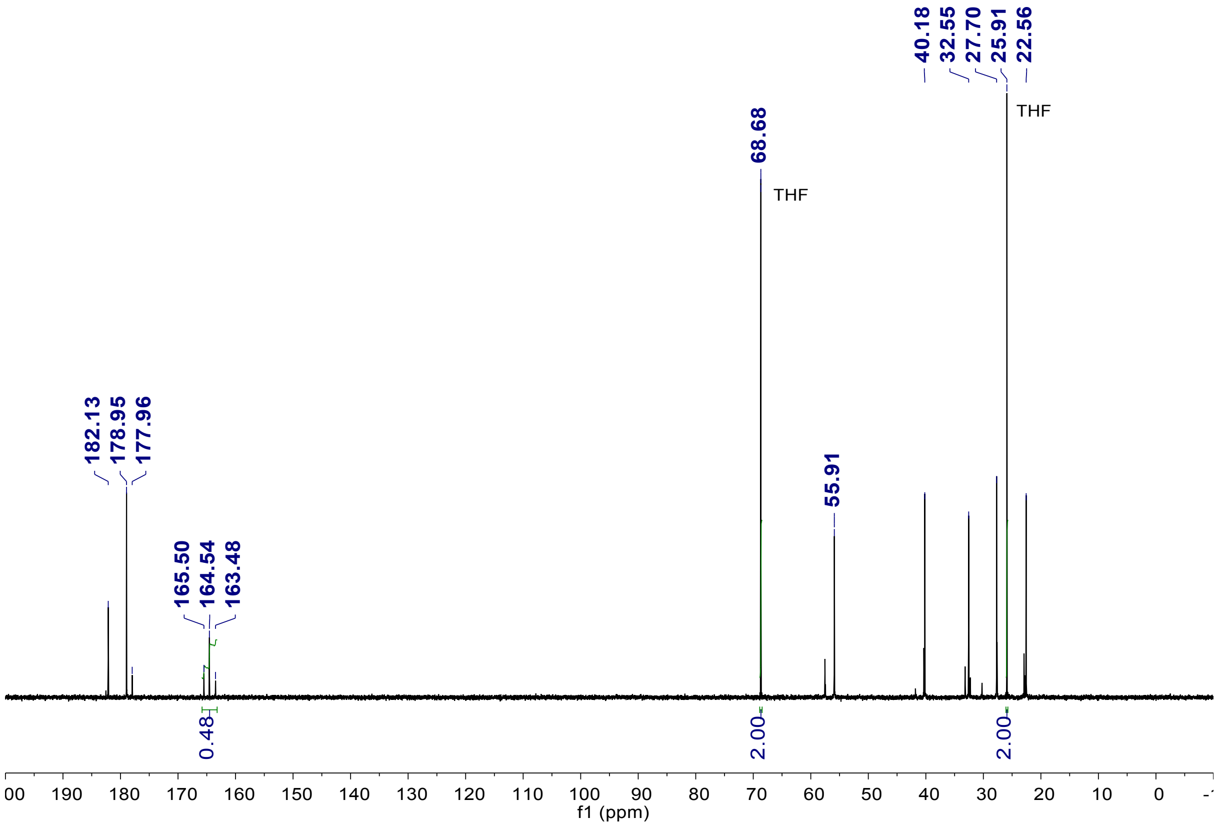

Figure S9. ^{13}C NMR-quant in D_2O of CO_2 capture with air bubbling (1 L/min. 4 days) with 5.0 mmol L-lysine.

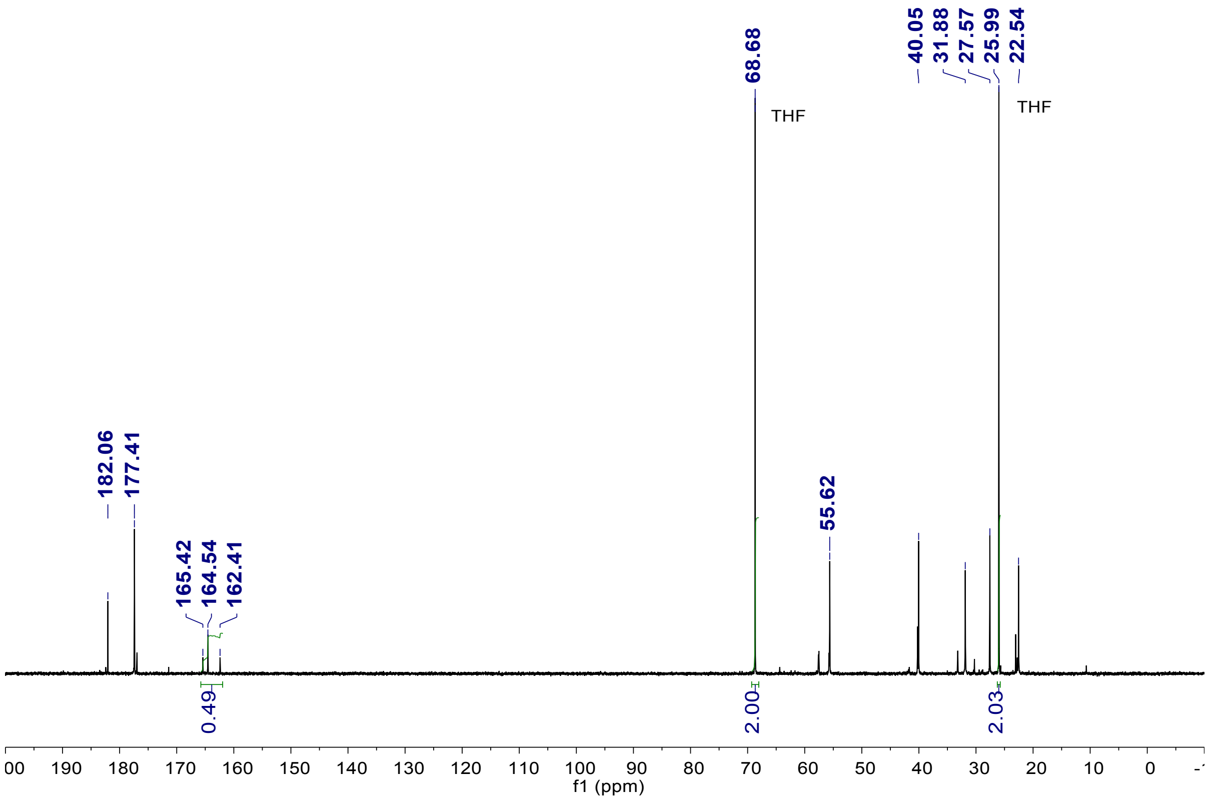

Figure S10. ^{13}C NMR-quant in D_2O of CO_2 capture with air bubbling (1 L/min. 8 days) with 5.0 mmol L-lysine.

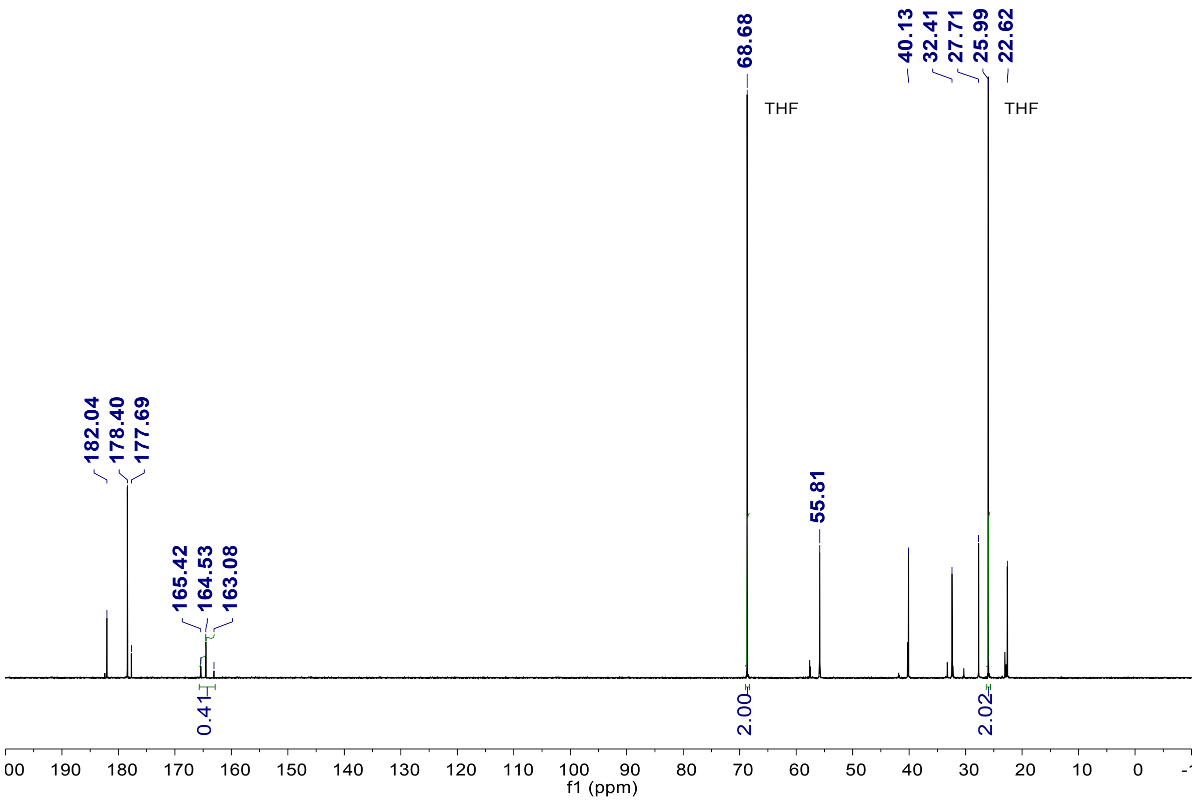

Figure S11. ^{13}C NMR-quant in D_2O of CO_2 capture with air bubbling (1 L/min. 4 days) with 20.0 mmol L-lysine.

Standard procedure for the hydrogenation of gaseous CO_2

Given amount of catalyst dosed from a stock solution (1 mg catalyst dissolved in 10 mL THF), amino acid (5.0 mmol) and solvent (10 mL) were added to a 50 mL autoclave equipped with a magnetic stir bar. After pressurizing the reactor with CO_2 gas, the reaction mixture was stirred at r.t. for 30 min. The reactor was pressurized with H_2 gas then heated and stirred on a pre-heated oil bath for indicated time. The reactor was cooled to r.t. and a biphasic reaction mixture containing a transparent upper layer and a yellow lower layer was obtained. DI water (ca. 3 mL) was added to the above mixture resulting in a homogeneous solution. DMF (250 μL, 3.24 mmol) was added as an internal standard to the reaction mixture. The reaction mixture was then analyzed by ^{1}H NMR with a few drops of D_2O (ca. 2 mL) to lock the signals.[4]

**5.0 mmol L-lysine**		
Before reaction (two-phase)	After reaction (two-phase)	After reaction (3 mL DI water added)
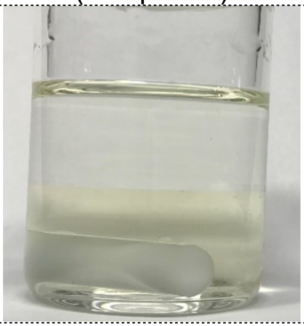	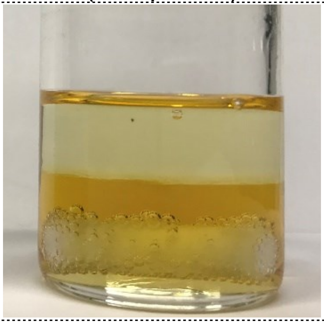	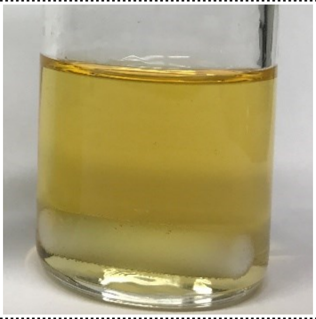
**20.0 mmol L-lysine**		
Before reaction (two-phase)	After reaction (two-phase)	After reaction (10 mL DI water added)
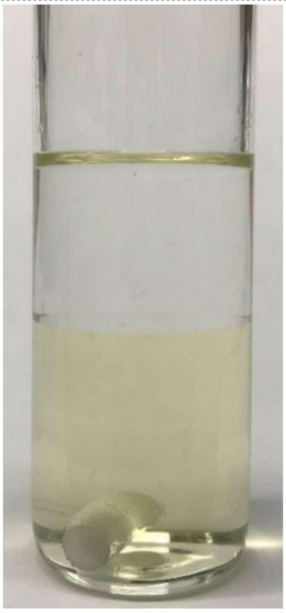	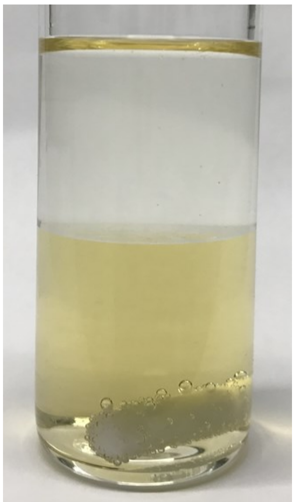	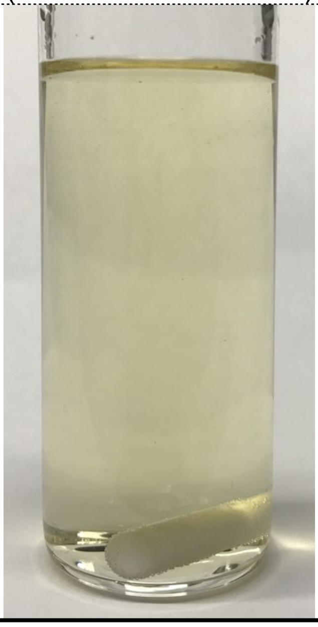

Figure S12. Typical reaction mixture of the hydrogenation of CO_2 to formate in the presence of L-lysine.

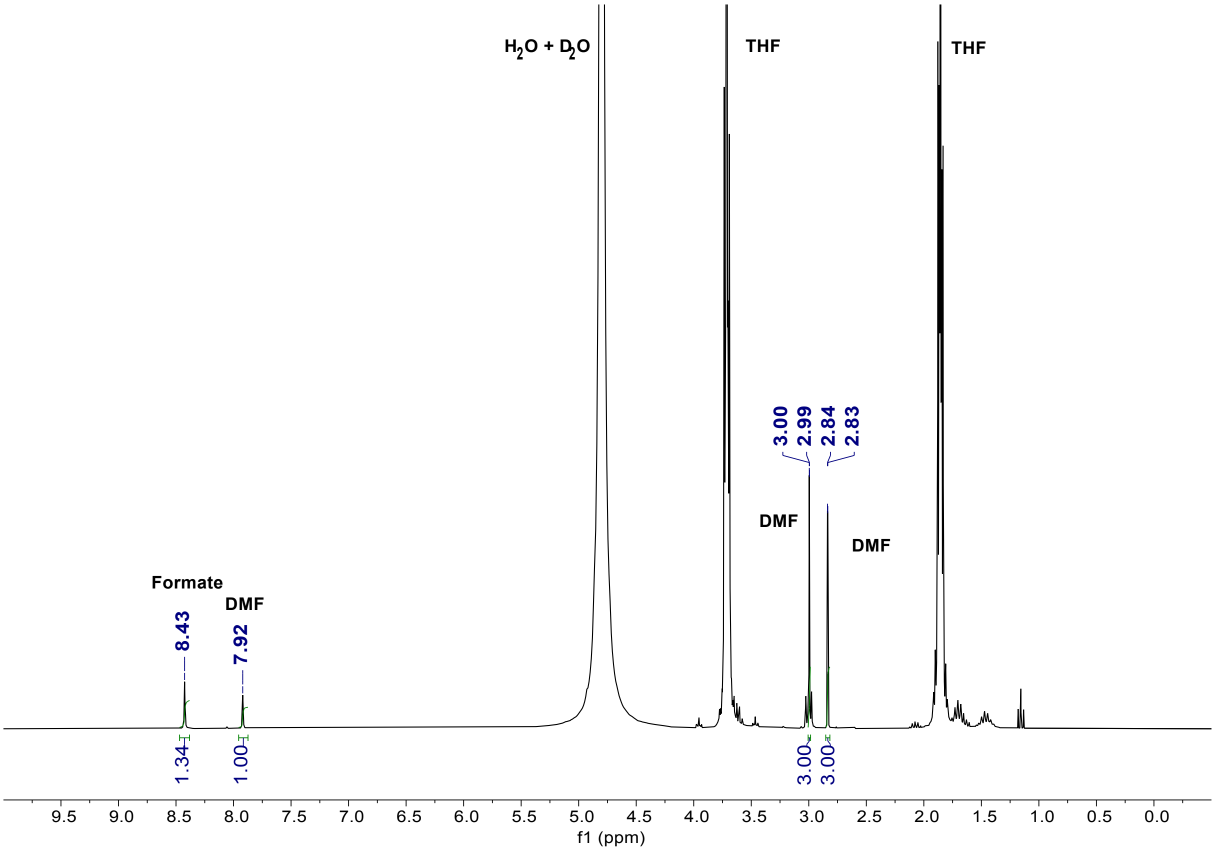

Figure S13. Typical ^{1}H NMR in D_2O after hydrogenation of gaseous CO_2 to formate in the presence of L-lysine.

Table S2. Hydrogenation of CO_2 in the presence of various amino acids.

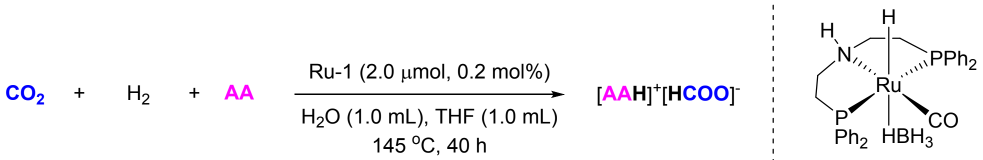

Ru-1
Ru-MACHO-BH

Entry	AAs		Formate [mmol][a]	Yield [%][b]	Formate [TON][c]
1	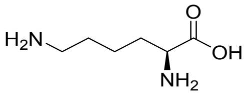	L-lysine[d]	0.71	71	355
2	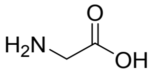	glycine	n.d.	-	-
3	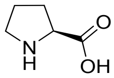	L-proline	n.d.	-	-
4	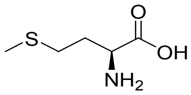	L-methionine	n.d.	-	-
5	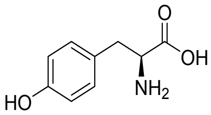	L-tyrosine	n.d.	-	-
6	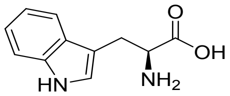	L-tryptophan	n.d.	-	-
7	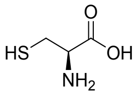	L-cysteine	0.04	4	20
8	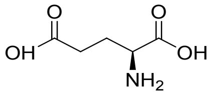	L-glutamic acid[d]	n.d.	-	-
9	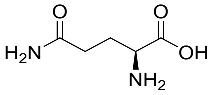	L-glutamine[d]	n.d.	-	-
10	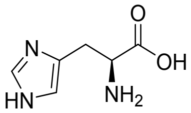	L-histidine[d]	0.125	13	63
11	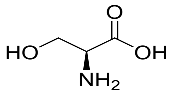	L-serine[d]	0.1	10	50
12	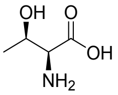	L-threonine[d]	0.045	5	23
13	None		n.d.	-	-

Conditions: AA (1.0 mmol), Ru-MACHO-BH (2.0 µmol, 0.2 mol%), H_2O (1.0 mL), THF (1.0 mL), CO_2 (20 bar), H_2 (60 bar), 145 °C, 40 h. [a] Determined by ^1H NMR with DMF (38.5 µL, 0.5 mmol) as internal standard. [b] Calculated by formate [mmol]/AA [mmol]. [c] Calculated by formate [mmol]/catalyst [mmol]. [d] AAs involved as RuBisCO active site. n.d.= not detectable. Experiments were performed at least twice; average values are used (St. Dev.<10%).

Table S3. Hydrogenation of CO_2 with L-lysine (blank reactions).

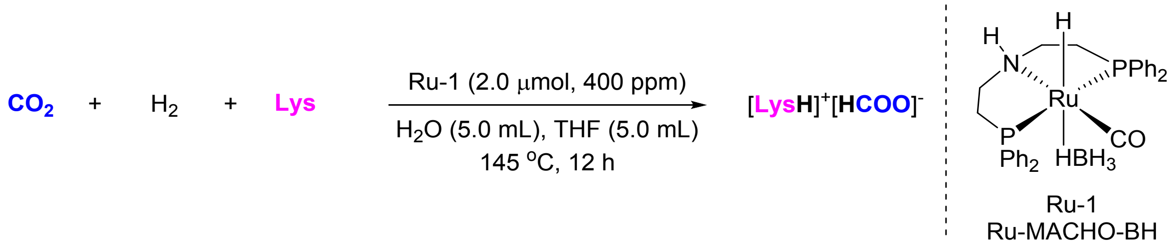

Entry	L-lysine [mmol]	Cat. [μmol, ppm]	Formate [mmol] [a]	Yield [%][b]
1	5	2.0, 400 ppm	4.37	87
2	None	2.0, 400 ppm	n.d.	-
3	5	None	n.d.	-
4 [c]	5	2.0, 400 ppm	n.d.	-

Conditions: L-lysine (5.0 mmol), Ru-MACHO-BH (2.0 μmol, 400 ppm), H_2O (5.0 mL), THF (5.0 mL), CO_2 (20 bar), H_2 (60 bar), 145 °C, 12 h. [a] Determined by ^1H NMR with DMF (250 μL, 3.24 mmol) as internal standard. [b] Calculated by formate [mmol]/L-lysine [mmol]. [c] In the absence of CO_2. n.d.= not detectable.

Table S4. Hydrogenation of CO_2 with L-lysine (screening of solvents).

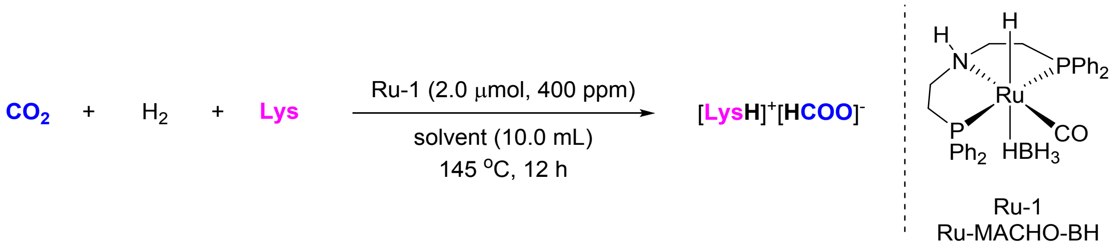

Entry	Solvent [mL]	Formate [mmol] [a]	Yield [%][b]	Formate [TON] [c]
1	THF [5] + H_2O [5]	4.37	87	2,187
2	2-MTHF [5] + H_2O [5]	4.30	86	2,148
3	Triglyme [5] + H_2O [5]	3.27	65	1,636
4	MeOH [5] + H_2O [5]	3.18	64	1,588
5	Ethylene glycol [5] + H_2O [5]	1.23	25	617
6	THF [10]	0.62	12	308
7	2-MTHF [10]	0.35	7	177
8	Triglyme [10]	0.55	11	275
9	MeOH [10]	0.52	10	259
10	Ethylene glycol [10]	1.13	23	567
11	H_2O [10]	0.49	9	243

Conditions: L-lysine (5.0 mmol), Ru-MACHO-BH (2.0 μmol, 400 ppm), solvent (10.0 mL in total), CO_2 (20 bar), H_2 (60 bar), 145 °C, 12 h. [a] Determined by ^1H NMR with DMF (250 μL, 3.24 mmol) as internal standard. [b] Calculated by formate [mmol]/L-lysine [mmol]. [c] Calculated by formate [mmol]/catalyst [mmol]. Experiments were performed at least twice; average values are used (St. Dev.<10%)

Table S5. Hydrogenation of CO_2 with L-lysine (screening of temperature and time).

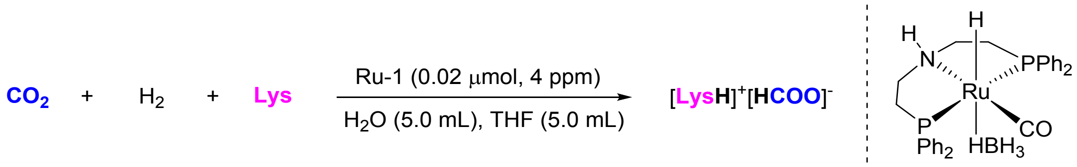

Ru-1
Ru-MACHO-BH

Entry	T [°C]	Time [h]	Formate [mmol][a]	Yield [%][b]	Formate [TON][c]
1	145	12	3.95	79	197,559
2	145	3	2.77	55	138,510
3	105	12	3.22	64	161,028

Conditions: L-lysine (5.0 mmol), Ru-MACHO-BH dosed from stock solution (0.02 µmol, 4 ppm), H_2O (5.0 mL), THF (5.0 mL), CO_2 (20 bar), H_2 (60 bar). [a] Determined by 1H NMR with DMF (250 µL, 3.24 mmol) as internal standard. [b] Calculated by formate [mmol]/L-lysine [mmol]. [c] Calculated by formate [mmol]/catalyst [mmol]. Experiments were performed at least twice; average values are used (St. Dev.<10%).

Table S6. Conditions for the generation of formamides from formates.

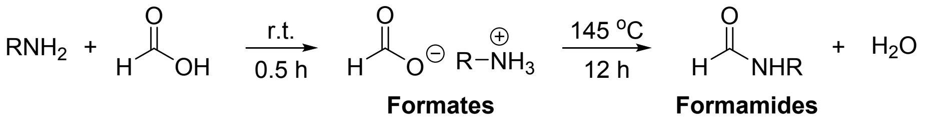

Entry	Amine [5 mmol]	Formates [%yield][a]	Formamides [%yield][a]	pH of free amine
1	L-lysine	>99	n.d.	10.2
2	PEHA	71	28	13.4

Conditions: Formic acid (5 mmol), L-lysine (5 mmol) or PEHA (5 mmol), H_2O (5 mL) as solvent, stirred at 145 °C, 12 h. pH of free amine was measured with 5 M concentration in H_2O at 25 °C. [a] Determined by ^{1}H NMR with DMF (250 µL, 3.24 mmol) as internal standard. n.d.= not detectable.

Standard procedure for CO_2 capture from ambient air and *in situ* conversion to formate.

The total volume of the solution of CO_2 capture from indoor air reduced to ca. 1 mL due to water evaporation. This mixture was firstly bubbled with argon for 30 min. then transferred using 4 mL of degassed DI water to a 50 mL autoclave equipped with a magnetic stir bar. The given amounts of catalyst (dosed from stock solution in THF) and THF (5 mL) were added to the above mixture. After pressurizing the reactor with H_2, the reaction mixture was stirred and heated on a pre-heated oil bath for indicated time. The reactor was cooled to r.t. and a biphasic reaction mixture containing a transparent upper layer and a pale yellow lower layer was obtained. DI water (ca. 3 mL) was added to the above mixture resulting in a homogeneous solution. DMF (250 µL, 3.24 mmol) was added as an internal standard to the reaction mixture. The reaction mixture was then analyzed by ^{1}H NMR with a few drops of D_2O (ca. 2 mL) to lock the signals.[4]

**5.0 mmol L-lysine**		
Before reaction (two-phase)	After reaction (two-phase)	After reaction (3 mL DI water added)
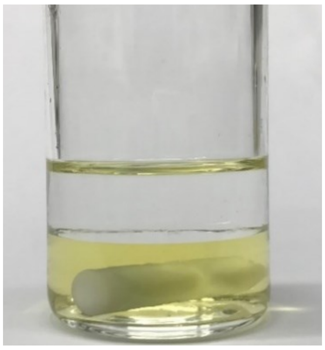	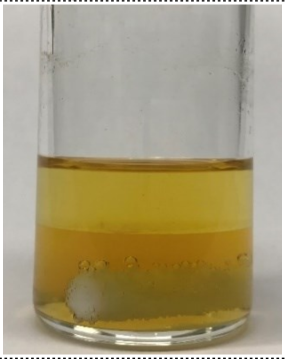	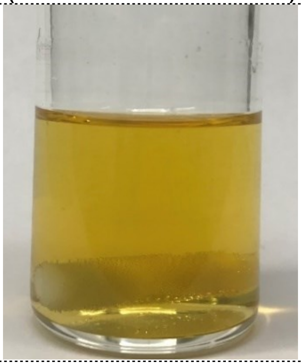
**20.0 mmol L-lysine**		
Before reaction (two-phase)	After reaction (two-phase)	After reaction (10 mL DI water added)
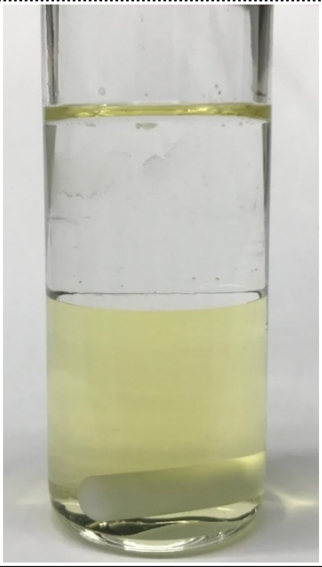	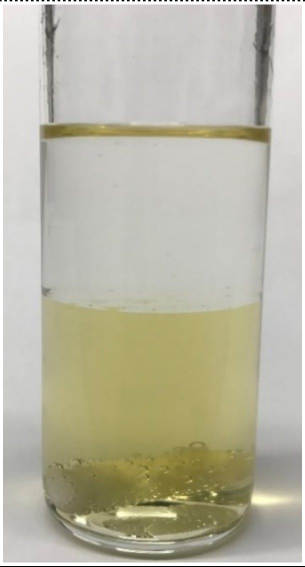	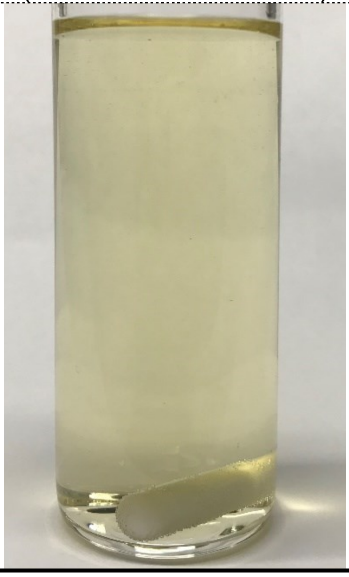

Figure S14. Typical reaction mixture for the hydrogenation of captured CO_2 to formate.

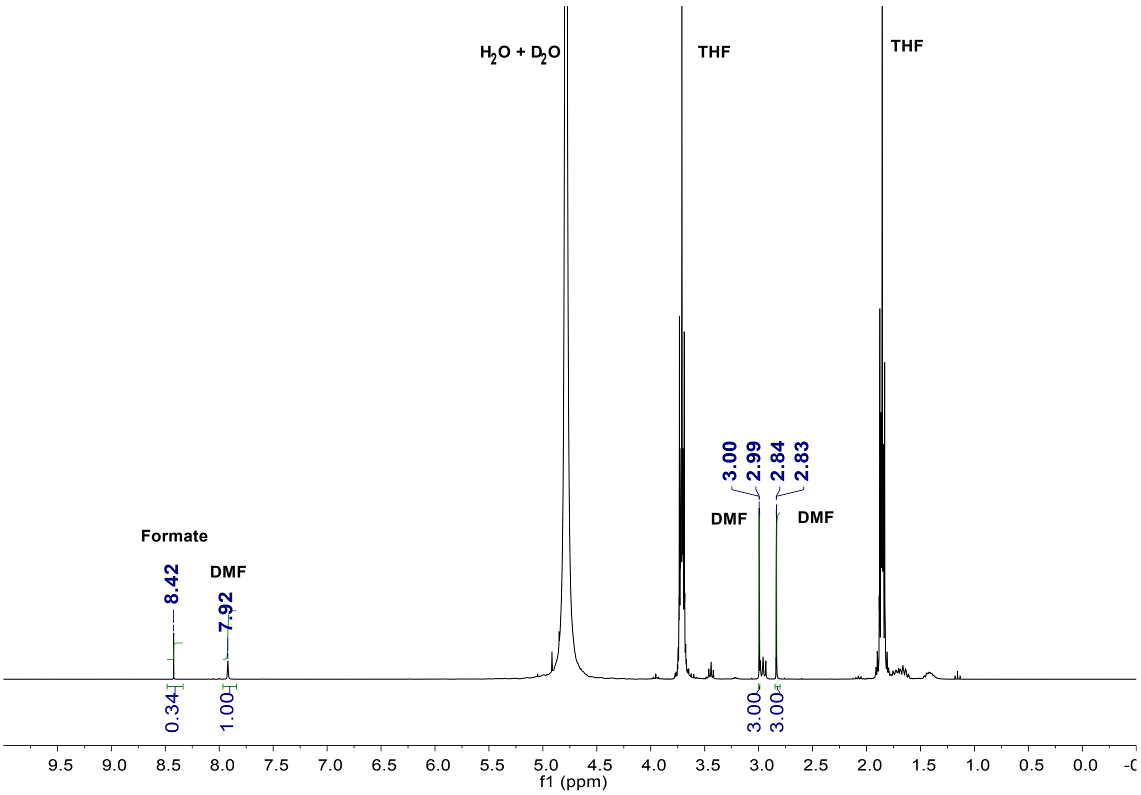

Figure S15 Typical ^{1}H NMR in D_2O after hydrogenation of captured CO_2 to formate.

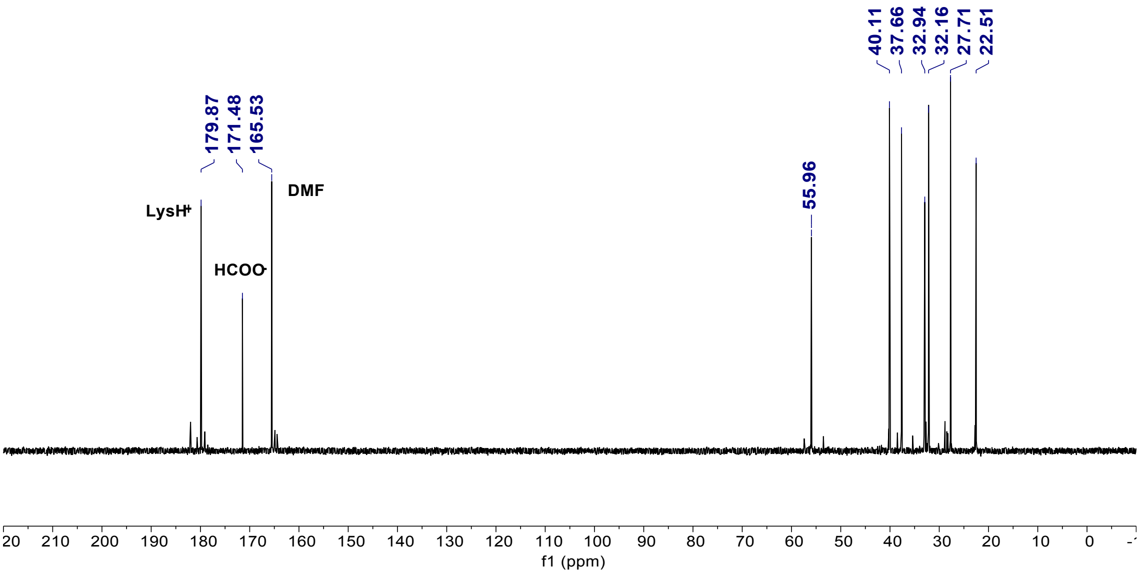

Figure S16. Typical ^{13}C NMR in D_2O after hydrogenation of captured CO_2 to formate.

Reference

(1) Gnanaprakasam, B.; Zhang, J.; Milstein, D., Direct Synthesis of Imines from Alcohols and Amines with Liberation of H_2. *Angew. Chem. Int. Ed.* **2010,** *49*, 1468-1471.

(2) (a) Alberico, E.; Sponholz, P.; Cordes, C.; Nielsen, M.; Drexler, H.-J.; Baumann, W.; Junge, H.; Beller, M., Selective Hydrogen Production from Methanol with a Defined Iron Pincer Catalyst under Mild Conditions. *Angew. Chem. Int. Ed.* **2013,** *52*, 14162-14166. (b) Werkmeister, S.; Junge, K.; Wendt, B.; Alberico, E.; Jiao, H.; Baumann, W.; Junge, H.; Gallou, F.; Beller, M., Hydrogenation of Esters to Alcohols with a Well-Defined Iron Complex. *Angew. Chem. Int. Ed.* **2014,** *53*, 8722-8726.

(3) (a) Barzagli, F.; Mani, F.; Peruzzini, M., A ^{13}C NMR Study of the Carbon Dioxide Absorption and Desorption Equilibria by Aqueous 2-Aminoethanol and N-Methyl-Substituted 2-Aminoethanol. *Energy Environ. Sci.* **2009,** *2*, 322-330. (b) Barbarossa, V.; Barzagli, F.; Mani, F.; Lai, S.; Stoppioni, P.; Vanga, G., Efficient CO_2 Capture by Non-Aqueous 2-Amino-2-Methyl-1-Propanol (AMP) and Low Temperature Solvent Regeneration. *RSC Adv.* **2013,** *3*, 12349-12355. (c) Perinu, C.; Arstad, B.; Jens, K.-J., ^{13}C NMR Experiments and Methods Used to Investigate Amine-CO_2-H_2O Systems. *Energy Procedia* **2013,** *37*, 7310-7317. (d) Kothandaraman, J.; Goeppert, A.; Czaun, M.; Olah, G. A.; Prakash, G. K. S., Conversion of CO_2 from Air into Methanol Using a Polyamine and a Homogeneous Ruthenium Catalyst. *J. Am. Chem. Soc.* **2016,** *138*, 778-781.

(4) Dubey, A.; Nencini, L.; Fayzullin, R. R.; Nervi, C.; Khusnutdinova, J. R., Bio-Inspired Mn(I) Complexes for the Hydrogenation of CO_2 to Formate and Formamide. *ACS Catal.* **2017,** *7*, 3864-3868.
